# A Research on the Classification and Applicability of the Mobile Health Applications

**DOI:** 10.3390/jpm10010011

**Published:** 2020-02-27

**Authors:** Ivan Miguel Pires, Gonçalo Marques, Nuno M. Garcia, Francisco Flórez-Revuelta, Vasco Ponciano, Salome Oniani

**Affiliations:** 1 Instituto de Telecomunicações, Universidade da Beira Interior, 6200-001 Covilhã, Portugal; goncalosantosmarques@gmail.com (G.M.); ngarcia@di.ubi.pt (N.M.G.); 2 Computer Science Department, Polytechnic Institute of Viseu, 3504-510 Viseu, Portugal; 3 Department of Computer Technology, Universidad de Alicante, P.O. Box 99, E-03080 Alicante, Spain; francisco.florez@ua.es; 4 R&D Unit in Digital Services, Applications, and Content, Polytechnic Institute of Castelo Branco, 6000-767 Castelo Branco, Portugal; vasco.ponciano@ipcbcampus.pt; 5 Altranportugal, 1990-096 Lisbon, Portugal; 6 Georgian Technical University, 0160 Tbilisi, Georgia; s.oniani@gtu.ge

**Keywords:** diagnosis, diseases, healthcare professionals, mobile applications, mobile computing, mobile health, treatment

## Abstract

Mobile health applications are applied for different purposes. Healthcare professionals and other users can use this type of mobile applications for specific tasks, such as diagnosis, information, prevention, treatment, and communication. This paper presents an analysis of mobile health applications used by healthcare professionals and their patients. A secondary objective of this article is to evaluate the scientific validation of these mobile health applications and to verify if the results provided by these applications have an underlying sound scientific foundation. This study also analyzed literature references and the use of mobile health applications available in online application stores. In general, a large part of these mobile health applications provides information about scientific validation. However, some mobile health applications are not validated. Therefore, the main contribution of this paper is to provide a comprehensive analysis of the usability and user-perceived quality of mobile health applications and the challenges related to scientific validation of these mobile applications.

## 1. Introduction

Nowadays, the use of mobile devices has increased, and this trend will continue in the coming years [1]. These devices make use of different platforms, but the two platforms responsible for the largest market share are the Android operating system (owned by Google) and the iOS operating system (owned by Apple) [2]. In general, technology is used to support the vast majority of users’ daily activities [1]. Due to their widespread integration with the users’ lifestyles, mobile devices can support personal activities anywhere at any time. In addition, these devices integrate numerous sensors that allow signal acquisition related to different aspects of medical or assisted living purposes in different environments [3,4,5,6,7,8,9,10,11].

Mobile devices integrate sensors and features that help healthcare professionals in the treatments of their patients with permanent connectivity. Mobile applications are useful for collecting data related to physical activity, human body images, and other aspects related to healthcare [12,13,14,15,16,17,18,19,20,21,22,23,24,25,26,27,28,29,30,31,32,33].

The increase of the complexity and extra processing associated with this type of mobile applications allows and increases the use of server-side data processing. Mobile applications or mobile apps are available for download over the Internet, in online application stores (e.g., Google Play and iTunes online store), with different categories [34,35]. 

Mobile applications help in the practice of health actions, clinical data collection, and health services. Moreover, these applications support the healthcare professionals to monitor the health state of their patients, check/share information, and perform diagnosis for several health problems [36,37]. The various areas for mobile health are “non-medical” applications critical for healthcare providers, drug reference, medical literature healing applications, medical calculators, medical language translators, general reference, patient education, specialty education, continuing medical education, physical and psychological analysis, health diagnosis, and sharing information over network, such as social networks [38]. Mobile health applications have issues related to learning rate, memorable rate, satisfaction, efficiency, and low error rate. In addition, one of the critical issues is the reliability assessment of mobile health monitoring systems [39,40].

The main contribution of this paper is to present the impact of mobile health applications for enhanced public health. The number of mobile apps related to the health topic is enormous, but several are not correctly validated and do not produce consistent results. Considering the diversity and the high number of these applications, it is not possible to analyze them all. Therefore, this document presents an analysis of several approved applications. These mobile applications are selected by following these four criteria: (1) developed for iOS or Android operating systems; (2) developed for the healthcare domain; (3) available for download in July 2019; and (4) available in other reviews or on the online application stores. Moreover, the mobile applications analyzed are selected according to the Google Play and Apple Store ranking. Other authors performed many analyses of mobile health applications but rarely analyzed scientific validation [38,41,42].

The healthcare professionals can use these applications to monitor the patients’ status, record or verify patients’ information, and consult medical prescriptions. The regulation of mobile health applications is made by the U.S. Food and Drug Administration, which defines the rules for the validity of mobile applications [43,44].

This paper introduces the concept of mobile health applications in Section 2. The relevance and impact of mobile applications in the overall health domain are presented in Section 3. Section 4 presents numerous features of these applications. Several taxonomies for mobile health applications are proposed in Section 5. Section 6 presents the conclusions of the study.

## 2. Concept of Mobile Health Application

More than 100,000 health mobile applications are currently available in the mobile store of Android and iOS. This number is anticipated to grow exponentially as technology and healthcare continue to develop side by side [45].

Mobile applications are software programs specially developed for mobile devices (e.g., smartphone, tablets, and smartwatch), which have a small size and resource consumption due to the features of these devices. Several types of sensors are available in mobile devices, including motion sensors, cameras, and proximity sensors. Mobile applications allow us to consult and analyze the collected data for various purposes. These tools can support the evaluation of clinical history, objective patient parameters, decision-making process data, communication with patients, and observing adherence to treatment [45].

Mobile health applications are software applications, related to health knowledge and research, used by health care professionals and patients to improve health treatments and public health. These applications transform a mobile platform into a regulated medical device. Mobile apps continue to displace older technologies in several domains, for example, by replacing applied communication devices for persons with autism and replacing medical bands with medical identification built into smartphone lock screens [46]. These applications use the data collected by sensors to handle the identification of various parameters related to patient health, such as physical activity, images of some part of a person’s body, and health state [47]. Furthermore, it is possible to recognize the vital parameters of people’s health using mobile sensors, allowing the recognition of daily activities and lifestyle [48]. Mobile applications help people to regulate or prevent some chronic diseases, such as obesity, diabetes, and cardiovascular accidents. Healthcare professionals use mobile health applications to improve and facilitate patient care. One benefit of mobile health, in conjunction with evidence-based practice (EBP), concerns its applicability to therapy preparation assignments [49]. Besides, medical apps are available for all types of useful functions such as electronic prescribing, assessment, clinical decision support, treatment practice management, coding, billing, self-care, and e-learning [50]. The apps are ready to help with the control of health-related habits such as diet, exercise, sleep, smoking cessation, relaxation, and medication adherence. They are prepared to aid the treatment of mental health conditions directly, such as anxiety disorders, depression, eating disorders, psychosis, and suicide prevention [50]. It is also necessary to involve public health agencies to provide all relevant information for patients experiencing from allergies and asthma in a daily and dynamically (ideally real-time) updated manner [51].

In general, mobile health applications have several purposes, e.g. diagnosis of cancer, diagnosis of heat rate problems, and controlling a glucose meter used by diabetic patients. However, a broad diversity of mobile health applications allows people to learn, improve, or share knowledge about healthcare, self-manage their diseases, and track health information. These applications also provide easy access to information related to health conditions or treatments; describe, show, or communicate potential medical conditions to their healthcare provider; automate simple tasks; and interact with electronic health record (EHR) or personal health records (PHR) systems. The mobile health applications provide rigorous and scientific evaluation and improve the empowerment of patients. Moreover, these applications provide bases of medicine and personal electronic records accessible over the Internet and promote the communication between healthcare professionals and patients. Mobile applications provide useful information to healthcare professionals and patients, enable information exchange, and enhance the cooperation between clinical units [52].

Mobile health applications provide numerous advantages related to electronic health processes, and most of them do not require an Internet connection. Mobile devices promote personalized health by providing user-friendly applications that can be used anywhere and at any time [53].

## 3. Relevance and Expected Impact of Mobile Health in the Overall Health Scenario

The number of mobile devices has increased, and, as a result, the number of mobile applications in online application stores, including Google Play and Apple online store, is growing [54]. Smartphones and mobile biosensors are multi-functional, low-cost, and have substantial general business penetration. Moreover, smartphones allow real-time psychological, behavioral, and physiological data [55]. The mobile health applications are widely successfully adopted by medical students, clinicians, and allied health workers for the fast dissemination of health news and critical health information [54]. Healthcare patients can also use mobile applications for leisure, sports practice, or other recreational activities. These applications are beneficial for people with chronic diseases or older adults to monitor their health state. Several studies already analyzed the clinical use of these applications [56]. These mobile applications use sensors to collect data related to the people, PHR, and other critical data over the Internet [43,44]. The applicability of mobile health applications lies in their capacity to enable chronic disease control and overall wellness motivation. By following a person’s health and raw lifestyle data, and by implementing actionable feedback, these tools support self-management. However, medical study and validation protocols are currently powerless to modify mobile health devices in useful time. Healthcare application development must be aware of the obstacles related to mobile health instruments, as well as the consequences of their responsibilities [57].

Sensors available on mobile devices can collect people’s data during physical activity. These data can be used to prevent health problems during sports, such as fatigue and cardiovascular issues [48]. These features have several benefits for healthcare professionals, such as for checking hearing, eyesight, and color recognition; evaluating the mental status; or capturing some information for future examination [54].

However, mobile health applications have some challenges, such as the requirement of network connectivity, battery power efficiency, usability, and data privacy [54]. The mobile health applications can help healthcare professionals to monitor the health state of their patients and the treatments of the diseases. They can also be used to track their patient’s location and improve the exchange of medical information using social networks [58].

For several chronic diseases such as Alzheimer’s and dementia care, the GPS (Global Positioning Systems) receiver can be used to control the location of these people [59]. The remote data capture at regular time intervals is useful for chronic diseases, such as diabetes, chronic obstructive pulmonary disease, and congestive heart failure [59]. Accelerometry sensors allow the recognition of other people’s physical activities, and this is relevant for older adults and people with some disorders [59].

The market for mobile health application will develop the following trends [60]:

- The smartphone user will be the main operator for the mobile health evolution.
- Mobile health applications will be tailored especially for smartphones, smartwatches, and tablets.
- Mobile health applications will be native applications rather than web-based applications.
- Mobile health stores will promote the second generation of mobile health apps.
- Lack of protocols and standards are the main challenge for mobile health applications.
- Mobile applications will enter common health distribution channels.
- Second-generation mobile health applications will focus on chronic diseases.
- Mobile health business models will increase.

Future mobile health business partners should promote multi-platform applications. Currently, more than 70% of mobile health app publishers choose both the iOS and Android platforms to present their applications [60]. As of 2017, there were around 259,000 medical applications in different online application stores, giving an unambiguous picture of the patient demand for these applications. According to Abdul Wahid, Lead Analyst at BIS Research, “Europe is the leading contributor to the global mobile medical apps market. The contribution of Europe to the mobile medical apps market was valued to be nearly 31.4% in 2016 and is growing at a compound annual growth rate (CAGR) of 29.7% during the forecast period. The rising number of government initiatives aimed at assessing the efficacy of medical apps for the benefit of public health may also contribute to the high growth rate. However, the Asia Pacific and Latin America are expected to grow at a higher CAGR than Europe i.e., 22.1%, and 23.1% respectively during the forecast period from 2017 to 2025” [61].

According to the results of Global Healthcare Information Software Market Report 2019 Analysis, Size, Share, Growth, Trends and Forecast 2019–2026, healthcare software marketplaces are mostly classified on the grounds of top advertising players, produce forms, applications, and global areas including North America, South America, Africa, and the Middle East, Europe, and Asia-Pacific. Healthcare Information Software Markets present market information about top manufacturers, market segmentation, types, application, and region. Healthcare Information Software Markets also shares market capacity, production, revenue, market drivers, and forecast opportunities and ambitions in the following years [62].

On the one hand, for healthcare professionals, mobile applications promote more agile clinical practices, helping diagnostics, monitoring, and prescription writing [56]. Healthcare professionals can also use mobile health applications to learn new health techniques, read health books, and communicate with their patients. On the other hand, for patients, mobile applications are a cost-effective approach to support disease treatment [53,63].

Excellent communication between healthcare professionals and patients anywhere at any time may minimize numerous health problems and diseases remotely [64]. Mobile health applications related to social networks allow proper communication between healthcare professionals and patients, exchanging information. They can share the experience of bodily symptoms, clinical diagnosis and treatment options, adverse treatment effects, sources of medical evidence, experiences with individual providers, and opinions about their quality [65].

Another purpose of mobile health applications is to help the treatment of psychological diseases. It involves direct and personal contact between patients and healthcare professionals to provide psychological support. Furthermore, Mobile health applications can be explored as mobile games that stimulate people to improve their metabolic health with physical activity and measure vital signs [66].

## 4. Mobile Health Applications and Classification

Mobile health applications are used for medical purposes, such as learning, treatment, diagnostic, and accomplishment, among others. However, these mobile applications are distributed by several categories at the online application stores for the utilization by different types of people, such as healthcare professionals and patients. This study analyzed several mobile health applications used by healthcare professionals and patients, for the Android and iOS operating systems. On the one hand, mobile apps used by healthcare professionals are about literature, patient monitoring and diagnosis, personal care applications, psychological health applications, educational applications, and social networking applications. On the other hand, mobile apps used by patients are individual care applications (e.g., fitness, sports, games, and auto diagnosis), application to check their PHR, apps to contact with their healthcare professional, educational health applications, and social networking applications.

The literature applications used by healthcare professionals in their studies, sometimes named as non-medical applications, are very useful for improving the treatments and knowledge about healthcare problems.

“Speed muscles MD” [67,68], “Speed bonnes MD” [69,70], “Speed Angiology MD” [71,72] and “Speed Anatomy quiz” [73,74] are paid mobile health applications, available for Android and iOS devices, focused on memory tests for the identification of the muscles, bones, arteries, and veins in the human body [54]. These applications are multilingual and primarily consist of obtaining the organ/bone that performs in the image. “Speed muscles MD” [67,68], “Speed bonnes MD” [69,70], “Speed Angiology MD” [71,72], and “Speed Anatomy quiz” [73,74] may be used to test the speed of reaction of the individual to perform specific actions, implementing a set of questions about anatomy and muscles with illustrative images on the theme.

“Medical Encyclopedia” [75] is a free mobile health application, available in Google Play and iTunes online store, which contains more than 50,000 pages of detailed medical information [54]. It can schedule an inquiry online, search for a medical specialist, and provide tests online.

“PubMed mobile pro” [76], a paid mobile health application available in Google Play store, and “Unbound MEDLINE” [77], developed for iOS devices, provide a responsive web interface to access the PubMed library [54]. These applications are used to search, read, store, and email articles related to individual health problems by author, journal, and relevant citations.

“Medscape” [78,79] is one of the most critical free mobile health applications in this category, developed for Android and iOS devices, offering a vast drug reference and a disease library, procedures, and protocols, and a drug interaction checker with more resources [54]. The key features are related to the access to the latest clinical news, interesting articles, safety information on more than 8500 prescriptions, and a network of doctors and medical students for consultation.

“MedCalc” [80,81] is a paid mobile health application, developed for Android and iOS devices, that provides easy access to a wide array of medical formulas and scores, including detailed information and bibliographic references for each formula [54]. It focuses on discovering new medical calculators, synchronizing individual data between web platform and automatic application, expecting content from use case authorities, and synopsis of evidence from original and validation research.

“Drug infusion” [82] is a paid mobile health application, developed for iOS devices, described as an intravenous medication drip rate calculator, with accurate calculations of dose, concentration, or rate of infusion, and offers both weight-based and non-weight-based predictions with unit conversion flexibility [54]. It is created for nurses and anesthetists to support the automatic calculation of drug dosage.

“Quick LabRef” [83] is a free mobile health application developed only for Android devices, which provides access to information on the most commonly used clinical laboratory values [54]. The mobile app provides access to laboratory data in several therapeutic areas, including detailed test protocols, rational of abnormal values, patient notes, and sophisticated graphic design.

“Medical Eponyms” [79,84] is a paid mobile health application, developed for Android and iOS devices, that allows for quick lookup of the meaning of more than 1700 medical eponyms using full-text search or by selecting from one of 28 categories.

“Taber’s medical dictionary” [85,86] is a mobile application developed for Android and iOS devices. This application presents a medical dictionary that contains more than 60,000 terms, 1000 photos, medical abbreviations, symbols and units of measurement, immunization schedules, and nursing diagnoses [54]. It is a multi-platform medical dictionary with useful appendices of laboratory values and alternative medicine plans.

“Sanford guide” [87,88] is a free Android and iOS mobile health application that allows the healthcare professionals who care for patients with infectious diseases to see their subscriptions [54]. It is a mobile application that provides information on contagious diseases, including clinical syndromes, pathogens, anti-infectious agents, specialized dosage tables and tools, calculators, and preventive therapy.

“Epocrates” [89,90] is a group of free mobile health applications, developed for Android and iOS for various purposes related to healthcare professionals [54]. The main features of these applications are related to drug prescribing and safety information, different forms, interactions between types of medications, tables of medications, calculators, and notifications.

“My pregnancy today” [91,92] is a free mobile health application, which supports 25 million women worldwide regarding their pregnancy questions available for Android and iOS devices [54]. This mobile application provides the monitoring of the state of the baby, calendar reminders and recommends physical exercises. Moreover, it includes checklists and turns possible to save photos and videos of the baby.

“WomanLog Calendar” [93,94] is a free mobile health application that shows a menstrual and fertility calendar for women, helping the women to know their fertile period [54]. The main features of this mobile application are the prediction or registration of the menstrual cycle, ovulation, and fertility, the tracking of the weight, the information about symptoms, mood and pills, and the reminders about different woman’s events.

“Pediatric Anaesthesia” [95], a paid mobile health application available for iOS devices, and “Pediatric Anesthesia” [96], developed for Android devices, requires the patient’s age, weight, and fasting time to help estimate the endotracheal tube size and give a maintenance rate [54]. It presents images with various tools for childcare related to multiple types of health problems, including breathing problems and glucose.

“ECG Guide” [97,98] is a paid mobile health application that consists of a teaching guide to Electrocardiography (ECG) interpretation [54]. It presents ECG images with different characteristics that represent various health conditions, where the healthcare professional can find in their patients.

“Normal Lab Values” [99,100] is a paid mobile health application, developed for Android and iOS devices, which shows reference values both in traditional and System International (SI) units [101]. This mobile application includes other features, such as organization, sorting, and localization of normal reference of laboratory values; commonly used clinical laboratory values; and other information related to the different tests.

“Medical Calculator” [102] is a paid mobile health application, available for Android and iOS devices, equivalent to “MedCalc”, which calculates the anion gap, bicarbonate deficit, calcium corrected for hypoalbuminemia, corrected sodium, glomerular filtration rate, creatinine clearance, fractional excretion of sodium, fractional excretion of urea and low-density lipoprotein cholesterol [101].

“Davis’s Laboratory and Diagnostic Tests” [103,104] and “Pocket Guide to Diagnostic Tests” [105,106] are paid mobile health applications, developed for Android and iOS devices, which provide evidence-based information on the procedure of common laboratory tests [101]. The main features of this mobile application are the availability of monographs about 450 laboratory tests and diagnostics; the adaption of the age, gender, and ethnicity variations; and the patient outcomes. In addition, this mobile application includes evidence-based recommendations, PubMed (PMID) links directly to citations of available journals and articles, search and favorites about the laboratory tests.

“Micromedex” [107,108] is a free mobile health application, developed for Android and iOS devices, used for on-the-go access to the industry’s most trusted clinical reference information, providing the peace-of-mind of knowing the information to the healthcare professionals. The main features of this mobile application are related to dosage information, side effects, drug interactions, administration of dosage adjustments, and generic drug names.

“Psych Drugs” [109] is a free mobile health application, developed for Android and iOS devices, that shows information about various psychotropic medications, including antipsychotics, anti-anxiety medications, medication for insomnia, mood stabilizers, depression, alcohol dependence, opioid dependence, and nicotine addiction for each drug. In Table 1, a summary of mobile health applications related to literature applications is presented.

Another category of mobile health applications used by healthcare professionals considered in this paper is related to patient monitoring to help the healthcare professionals in monitoring their patients. “TeleCardio” [110] is a free mobile health application, developed for Android, for remote monitoring of patients with heart conditions with built-in mechanisms for analysis of ECG signals and generation of automatic alerts in case of emergencies [111]. This mobile app includes features such as a notebook to store contacts, dates of appointments, and information about treatments. Additionally, it consists of a travel guide with the recommended precautions and the list of countries where the app works.

“MedKart” [112] is a free mobile health application, developed for Android, providing access to the hospital information system (HIS) and picture archiving and communication system (PACS). It is a useful application for medical equipment acquisition [111].

“AirStrip ONE” [113,114] is a free mobile health application, developed for Android and iOS devices, connecting patients to healthcare professionals anywhere, reducing and eliminating the time delay in the clinical assessment and treatment [41]. These mobile health applications, based on telemedicine and health at a distance, can be accessed with mobile devices to improve the people’s health and monitoring anywhere at any time. This mobile application allows the doctor to view the output of clinical examinations on the mobile phone.

The mobile health applications can help healthcare professionals in the diagnosis and treatment of their patients, and this category is named “diagnosis applications”. “iTriage” [115] is a free mobile health application, developed for Android and iOS devices, that is related to the diagnosis of the patient’s health state and finds a healthcare professional in their location [116]. The main features are related to contacting emergency medical services in the case of health problems, seeking medical treatment or a doctor, checking their symptoms, and searching for disease information and procedures for clinical therapies.

“Diabetes Buddy” [117] is a paid mobile health application, developed for iOS devices, that helps people to manage diabetes; track factors that influence the blood sugar level, including glucose, medication, activity, water consumption, and weight; monitor the fluctuations of blood sugar level; and plan and assist people in sharing these data with their healthcare professionals [116].

“Glucose Buddy” [118,119] is a free mobile health application, developed for iOS devices, that tracks glucose readings entered four times a day, as well as food consumed, exercise, insulin dosage, and activities and allows sending this information by e-mail [42]. The main features of this mobile application are related to the logging of blood glucose, medication, and meals, and the tracking of trends in blood sugar, insulin, weight, and blood pressure. Besides, it allows users to add notes to the entries for future reference, observe the changes in blood sugar and carb intake, and log the meals using an extensive food database.

“HelpDiabetes” [120] is a free mobile health application, developed for Android and iOS devices, that calculates the total carbohydrates, fats, and proteins of ingredients [42]. This mobile application allows the tracking of blood glucose levels, insulin, and other medication; the calculation of active insulin based on own configurable insulin profile; the monitoring of sport and other activities; and the creation of own food composition table.

“Diabetes App—blood sugar control, glucose tracker, and carb counter” [121] and “Diabetes Lite App—blood sugar control, glucose tracker, and carb counter” [122] are mobile health applications, developed for iOS devices, which were built to help patients manage their diabetes by controlling their activities and exercises, tracking the factors that influence their blood sugar level, monitoring the fluctuations, planning accordingly and sharing their data with their healthcare professional. In Table 2, a summary of mobile health applications related to diagnosis and treatment is presented.

Healthcare professionals can use mobile applications related to personal care, and it has a high variety of mobile apps, e.g., caloric counter, control the fitness and sports, auto-diagnosis, and games. “MyPlate” [123,124] is a free mobile health application, developed for Android and iOS devices, that is related to the control of the user’s diet, weight change, and workout to help people stay fit, including a calorie counter that compares the calories spent to the calories that the user consumed eating [116]. The main features of this mobile application are related to the visualization of a food database and the use of a bar code scanner to find and track food. Additionally, other features are present, including the creation of custom foods and meals, the personalized daily calorie goal based on your profile information, the tracking of weight and progress over time, and the nature of daily custom goals for the nutritional intake of protein, fat, carbs, fiber, sugar, and sodium. Finally, it allows the tracking of water intake, the setting of meal-time reminders, the integration with Google Fit, the logging of workouts, the creation of custom exercises, and the insertion of calories burned.

MyFitnessPal” [125,126] is a free mobile health application, developed for Android and iOS devices, that helps with the user’s diet, controlling your weight and calories spent during your daily physical activities, storing the results in a remote database [127]. Many functionalities are available in this mobile application, including a food database, barcode scanner, recipe importer, restaurant logging, food insights, calorie counter, macronutrient and water tracker, creating a diet diary, customizing goals and habits, and logging exercise and steps.

“Weight Watchers” [128,129] is a free mobile health application, developed for iOS devices, which calculate the calories needed and uses a barcode to insert the items and check your calories, using a database of over 250,000 foods. Complementary, this mobile application allows monitoring the fitness goals with an activity tracker.

“iMapMyRIDE fitness GPS” [130,131] is a free mobile health application, developed for iOS devices, that measures the calories spent, the time, and separating these values by types of activities. The main features of this application are the connection with other mobile applications and wearables, and the existence of a community to meet other users of the app and control the user’s nutrition.

“Fooducate” [132,133] is a free mobile health application, developed for Android and iOS devices, which monitors the weight loss and diet control. This mobile application allows the tracking of meals, food intake, and exercise; the quality of calories and macronutrients; the integration with other mobile apps; the addition of the recipes; and the availability of a food database with calories.

“Mindful Eating” [134,135] is a paid mobile health application, developed for iOS devices, that helps to build mindful eating habits over time with the award of badges for nutritional milestones and points of dietary facts about food. Finally, it recognizes patterns and provides tools for changing the user’s diet [42]. The mobile application helps users choose food, encouraging messages, see progress through the resize screen.

“Tap and Track” [136] is a paid mobile health application, developed for iOS devices, which focuses on food, exercise, and weight, computing the nutritional intake on foods eaten, physical activity, current weight, and target weight; tracking the calories spent by calculating basal metabolic rate (BMR); and finding daily calorie count based on gender, age, weight, height, and the lifestyle [42]. Using this mobile application, the user can track food and burned calories; control daily weight; set diet plan, goal weight, fat budget, carbs budget; and view body mass index (BMI) and nutrition data, e.g. calories, protein, carbohydrate, fat (and saturated fat), fiber, sodium, and sugar.

“Is That Gluten-Free?” [137,138] is a paid mobile health application, developed for iOS devices, designed for those with gluten sensitivities, celiac disease, or anyone interested in gluten-free eating, including searching by categories, brand, and product name and filtering by safe, unsafe, and possibly unsafe ingredients [42].

“Cook IT Allergy Free” [139] is a paid mobile health application, developed for iOS devices, that provides a library of recipes for those sensitive to gluten, dairy, eggs, and nuts, suggesting substitutions and offering the ability to customize methods to avoid specific ingredients and add personal recipes [42].

“Diet and Food Tracker” [140] is a paid mobile health application, developed for Android devices, used to track foods, calories, and weight, and it can follow some exercises. This mobile application includes a database with over 3,500,000 foods tracked, monitoring of diet, fitness, and weight, availability of diet programs agnostic, calculation of food intake and nutrition, integration with other mobile applications and devices, and demonstrations of physical exercises.

“Full Fitness” [141] is a paid mobile health application, developed for iOS devices, that provides an exercise database with bright illustrations, videos, and instructions, allowing the users to add exercises, track their progress, and e-mail the data [42]. Thus, this mobile application includes a database with unique activities, images related to the practices, video instructions, tracking of calories of over 90,000 food items, logging and monitoring of different exercises, a chart with workout details, weight monitoring, calculation of BMI, and support to sharing in social networks and cloud.

“GymPact—Absolute Fitness App” [142] is a paid mobile health application, developed for Android devices, that allows users to log and manage their food intake, exercise, and weight, allowing the automatic calculation of daily nutrient limits based on personal profile and dietary goals [42]. In addition, it enables increasing weight loss, tracking GPS receiver, accelerometer and step counter, and the connection with other mobile applications and devices.

“Quit IT—Stop Smoking Today” [143] is a paid mobile health application, developed for iOS devices, which motivates and encourages to quit and helps ex-smokers avoid relapse. For the purpose, it tracks the number of cigarettes not smoked and checks the money saved. To achieve these purposes, it allows the definition of daily goals, showing the quantity of the nicotine and carbon monoxide not consumed and the benefits of quitting [42].

“Quit Smoking-Cold Turkey” [144] is a paid mobile health application, developed for iOS devices to help the user to stop smoking, with the calculation of the number of cigarettes [145]. This mobile application allows the tracking of the number of times with the desire to smoke, the setting of a date to stop smoking and the progress sharing. In Table 3, a summary of mobile health applications related to personal care applications is presented.

Another category of applications used by healthcare professionals is mobile applications for psychological purposes. “Awareness” is a paid mobile health application, developed for iOS devices, which is a tool to intercept peoples’ daily routines and prompt them to get in touch with what they are feeling. The feelings are created into the present moment, providing insight and breaks patterns of emotions, attitudes, and behavior through awareness and inspirational practices [42]. For different purposes, this mobile application has 20 meditative music videos, 400 inspirational quotes, and daily, weekly, monthly, and yearly reports that provide information about your mood, feelings, and associated activities.

“Meditation Timer” [86,146] is a paid mobile health application, developed for iOS devices, used to listen to meditations that can be customized by name, time, and sound settings [42]. It also allows setting session timers, alarms to access the mobile application, the duration of sessions, background sounds for meditation, and the automatic rejection of incoming calls during sessions.

“Relax Melodies” [147,148] is a free mobile health application, developed for iOS devices, that offers 70 high-quality ambient sounds, volume adjustment for each sound, and binaural beats for brainwave synchronization to help induce relaxation, guided meditation, sleep medications, and breathing techniques [42].

“Zen Timer” [149,150] is a paid mobile health application, developed for iOS devices, used for mindful activities, such as meditation and yoga, playing sounds, and allowing the connection with social networks [42]. It is a cross-platform mobile application to set meditating tools with critical features, such as creating and editing of meditation schedule, setting reminders for the user to practice meditation, and supporting the transfer of data to health app.

“Simply Being-Guided Meditation for Relaxation and Presence” [151] is a paid mobile health application, developed for iOS devices, used to choose voice-guided meditation, with a length of 5, 10, 15, or 20 min with the option to listen with or without music or nature sounds [42]. The user can able choose the time that the music or nature sounds continue after the meditation session ends.

“Bodhisattva Mind Teachings to Cultivate Courage and Awareness in the Midst of Suffering by Pema Chodron” [152] is a paid mobile health application, developed to iOS devices, that explores the insights and practices from the teachings of an eighth-century Buddhist classic, The Way of the Bodhisattva, the key to true liberation and the taming of the mind [42].

“Hatha Yoga-Your Portable Yoga Studio” [153] is a free mobile health application, developed for Android devices, that provides hours of professionally recorded instruction, including built-in classes for various skill levels, the ability to customize any type to suit individual needs, meditation and breathwork education, and the option to select the background music [42]. This mobile application is also able to show videos related to yoga and meditation classes, show 280 poses with detailed advice and instructions, and allow the tracking of fitness goals.

“Yoga Relax” [154] is a paid mobile health application, developed for iOS apps, that shows yoga sessions, including the ability to turn on or off an audio instructor, detailed information about poses, the required steps for correct positioning, and how to maintain a pose [42]. This mobile application is similar to “Hatha Yoga-Your Portable Yoga Studio”.

“Authentic Yoga with Deepak Chopra and Tara Stiles” [155] is a paid mobile health application, developed for Android and iOS devices, that contains video explanations of individual poses, breathwork, and other yoga practices with customizable routines [42]. In Table 4, a summary of mobile health applications related to psychological purposes is presented.

Other mobile applications are related to education for healthcare professionals. “Electronic Preventive Services Selector (ePSS)” [158] is a free mobile health application, developed for iOS and Android devices, that is a set of calculators and tools with simple screening tools for a variety of situations [38]. The information is related to the current US Preventive Services Task Force (USPSTF) recommendations, and it is available for patient-specific characteristics such as age, gender, and selected behavioral risk factors.

“Radiology 2.0” [159] is a free mobile health application, developed for iOS devices, that contains 65 radiology cases that pertain to emergency medicine [38,54]. It is used to teach the user to analyze the computed axial tomography using didactic files. In Table 5, a summary of mobile health applications related to education for healthcare professionals is presented.

In some cases, patients need direct contact with their healthcare professionals. Mobile health applications can improve communication between patients and healthcare professionals. This category contains the mobile health applications related to telemedicine, but this area includes a few other mobile apps that enhance the collection of some sensors signals during the contact with their healthcare professional.

“WebMD: Check Symptoms, Find Doctors, & Rx Savings” [160,161] is a free mobile health application, developed for Android and iOS devices, specially designed for people with head pain, neck pain, nerve pain, fibromyalgia or migraine, allowing patients to control their lifestyle choices, review personal patterns to understand triggers, set goals, and easily share progress with their healthcare professional,.

Patients can also check their PHR anywhere at any time with mobile health applications. “Capzule PHR” [162], “Health and Family” [163], and “HealthNotes” [164,165] are paid mobile health applications developed for Android and iOS devices that allow patients to check and organize their medical information and access to their PHR anywhere and any time over the Internet and has offline access [166]. “Capzule PHR” [162] also allows viewing profile, reminders, and appointments; creating summary in PDF; creating custom health monitoring templates to track any chronic condition; setting medication rand doctor appointment reminders; creating QR code summary for emergency personnel; recording conditions and allergies; tracking health statistics with flowsheets and self-health screening; writing notes; and managing physician and insurance information. “Health and Family” [163] and “HealthNotes” [164,165] are used to take health notes and message doctors.

“OnPatient Personal Health Record” [167] is a free mobile health application, developed for iOS devices, that allows the patient to access their PHR anywhere at any time with security by Internet or offline in the mobile application [166].

In general, these applications allow access to the patient to their PHR. “Patient” [168,169] is a free mobile health application, developed for Android and iOS devices, that allows the patients to access the latest news, publications and health information; book appointments; order repeat prescriptions; explore local pharmacy services; and access their medical records. In Table 6, a summary of mobile health applications to check PHR is presented.

Patients can use mobile health applications to learn the primary news and activities of healthcare problems, the human body, and first-aid applications. “drawMD - Patient Education” [170] is a free mobile health application, developed for iOS devices, related to the patient education and contact between patients and healthcare professionals [54]. This group of applications contains mobile apps related to cardiology, general surgery, orthopedic surgery, obstetrics, gynecology, urology, and anesthesia [54].

“First Aid” [171,172] is a free mobile health application, developed for iOS and Android devices, that provides information on urgent and emergent medical situations [38]. This mobile application includes a variety of treatments, such as allergic reaction, asthma attack, bites and stings, burns, chest pain, choking, defibrillation, diabetic emergency, fractures, recovery position, removal of the helmet, severe bleeding, shock, sprains, and strains.

“VueMe” [173] is a free mobile health application, developed for iOS devices, used by patients for the non-diagnostic viewing of medical images and share it to a cloud service. It also allows the real-time multi-modality fusion and blending, 3D depth-shaded movie, and secure and encrypted connections.

“iMuscle 2” [174] is a free mobile health application, developed for Android and iOS devices, used to identify a body part or individual muscle by zooming into a 3D human body with the musculature exposed and access to all the exercises associated with the development or rehabilitation of that muscle. The most important features of this mobile application are the rotation and zooming-in to the real 3D model with the musculature exposed to reveal superficial and many deep muscles. Other features available are the availability of high-quality 3D animated images of exercises, searching activities by muscles, creation of exercise plans, tracking user progress, and sharing of statistics in social networks. Table 7 summarizes mobile health applications related to educational applications for patients.

The last category of mobile health applications used by healthcare professionals presented is social networking applications for health purposes. “Doximity” [175,176] is a social network that has a mobile health application, developed for iOS and Android, which is a more comprehensive peer-to-peer communication toolkit that includes a global search function to help the health care professionals to find another for free communication, Health Insurance Portability and Accountability Act (HIPAA) compliant e-fax tool, direct messaging feature, and healthcare professionals’ community, typically formed around shared interests.

“Univadis US” [177,178] is a mobile health application, developed for iOS and Android devices, that is a community of healthcare professionals organized around the general aim of learning and improving their practice and users can directly message other users outside of public forums, with interactions around topics in medical research, clinical care, policy, and regulation [38]. This mobile application allows collaboration.

“DocBookMD” [179,180] is a free mobile health application, developed for Android and iOS devices, that allows the communication between healthcare professionals; they can send X-rays, EKGs, and other patient’s information directly to another colleague, enabling fast, secure, and HIPAA-compliant multi-media messaging to enhance patient care [181]. This mobile application includes the ability to send and receive HIPAA-secure messages and images, invite coworkers to be part on DocbookMD, attach pictures straight from the device camera, and alert users from an urgent communication and easy integration with the laboratories. Table 8 summarizes mobile health applications related to social networking.

Several mobile health applications are available in the online application stores, and these applications are a result of studies in universities or research companies. The mobile health applications presented in this section are the applications with more downloads and more citations in other research. The mobile apps meet the regulations defined for the validity of mobile applications related to healthcare. Due to the importance of this area of mobile applications, the scientific validation of these applications sustained by laboratory research studies is essential. These applications are critical to improving people’s health with equipment commonly used. The mobile apps presented are the ones with better rank in the stores or cited in different scientific studies. The applications presented in this paper are validated and approved by the users or supported by scientific studies. In general, the mobile health applications are free of charge, or the user only needs pay to access to other functionalities, which are not the main functionalities of the mobile apps. However, the more technical applications are always paid, due to the costs of the research performed. Medical communities use a large part of the mobile apps presented in this paper.

## 5. Discussion

Following the classification and analysis performed, there are many advantages and disadvantages related to the use of mobile apps in the diagnosis, prevention, and treatment of different diseases. Thus, the classification proposal includes mobile applications that are the most ranked by users. People of different ages can install and use these software components, but the most critical functionality consists of the dissemination of crucial information about various diseases. The major problem of these mobile applications is the dependence on the Internet connection to provide updated information. Currently, the data connection is available in different hotspots, residences, and hospitals, allowing the correct use of these applications. However, personal contact is always essential, and these applications cannot replace human care. Mobile applications also allow contact with a doctor, improving the treatment of several diseases. 

**Figure 1:**
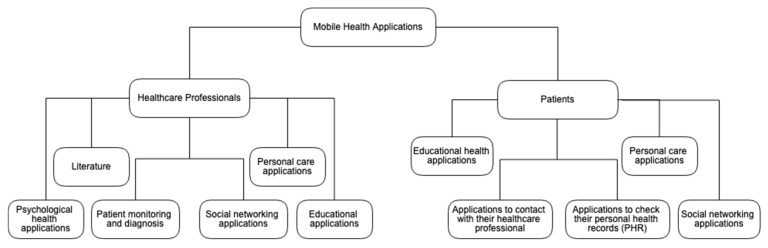
Classification of mobile health applications studied.

In mobile operating systems, the applications are commonly grouped according to their functionalities. Therefore, mobile health applications used by healthcare professionals and their patients have been classified by the authors based on their functionality (Figure 1). These mobile applications analyzed in this paper classified into seven groups: literature, patient monitoring, diagnosis, personal care, psychological health, educational applications, and social networking applications. According to the analysis of the mobile applications included in each category, the mobile app has different features, and most of them require a constant network connection. Several of these applications analyzed are only available for the iOS operating system. Moreover, the most reliable apps are paid or contain in-app purchases to access different functionalities.

**Figure 2:**
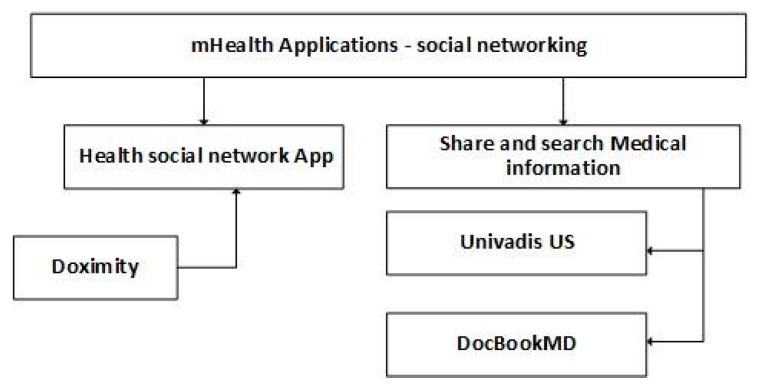
Classification of mobile health applications related to social networking.

Figure 2 presents the classification of mobile health applications related to social networking. These applications allow sharing and searching medical information. It is the category of mobile health applications that increases the possibilities to share different information rapidly. However, this information is not validated and accepted by medical communities, considering the possibilities of sharing invalid information, also known as “fake news”. 

**Figure 3:**
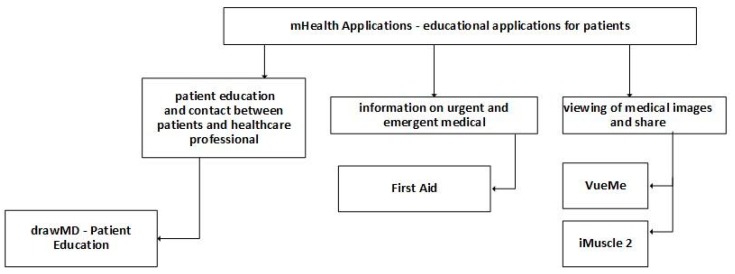
Classification of mobile health applications related to patient education.

The education of the patients plays a significant role to filter “fake news” from correct information. Figure 3 divides these mobile apps into three types: mobile applications that provide the communication between patients and healthcare professionals, mobile applications that provide the sharing of urgent and emergent medical information, and mobile applications that provide the use of educational images to teach different types of patients. These mobile applications help to inform the patients about the various procedures that should consider in case of specific symptoms.

**Figure 4:**
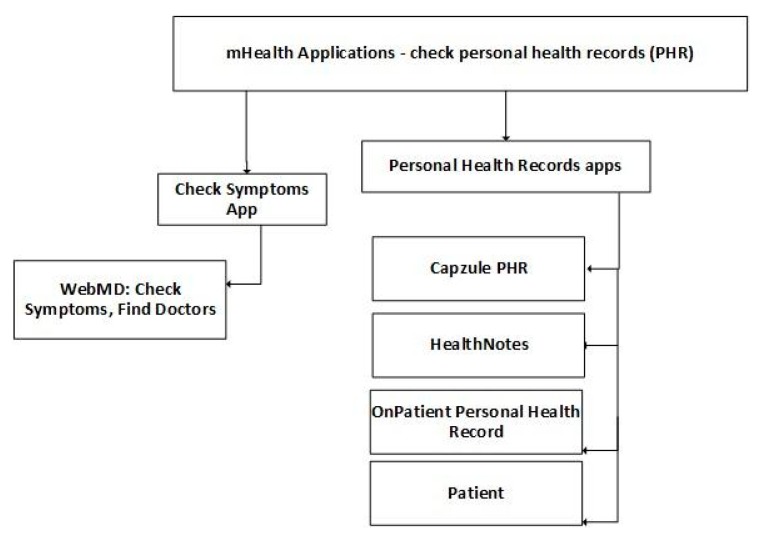
Classification of mobile health applications related to PHR.

Another relevant functionality is the possibility to consult PHR, as presented in Figure 4. These mobile applications can be divided into mobile apps which enable the consulting of symptoms and PHR. The most crucial point of these applications is the security of information accessibility as this information contains relevant patient’s data. Security is a significant challenge related to the lower acceptance of mobile applications in medical communities. Therefore, mobile applications should be validated and tested before their availability in the market.

**Figure 5:**
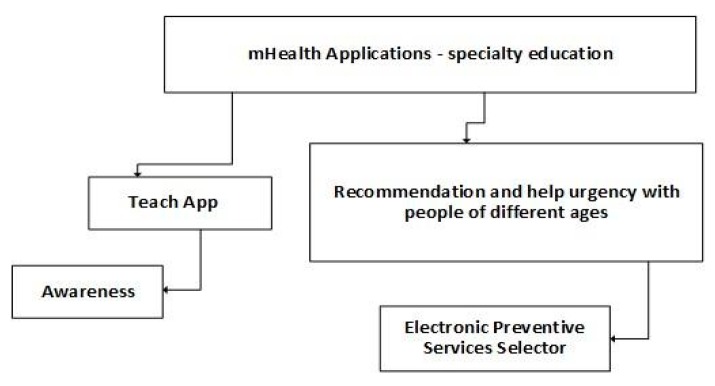
Classification of mobile health applications related to specialty education.

People with special needs require monitoring, teaching, and constant help to improve their quality of life. Thus, Figure 5 proposes the classification of the analyzed mobile applications related to specialty education. The mobile software includes applications for teaching purposes and to deliver recommendations in urgent situations. The adaption of the different needs for training is one of the most important things of these mobile applications. However, it causes the necessity of constant Internet connection, and there is no scientific validation of these applications before their use.

**Figure 6:**
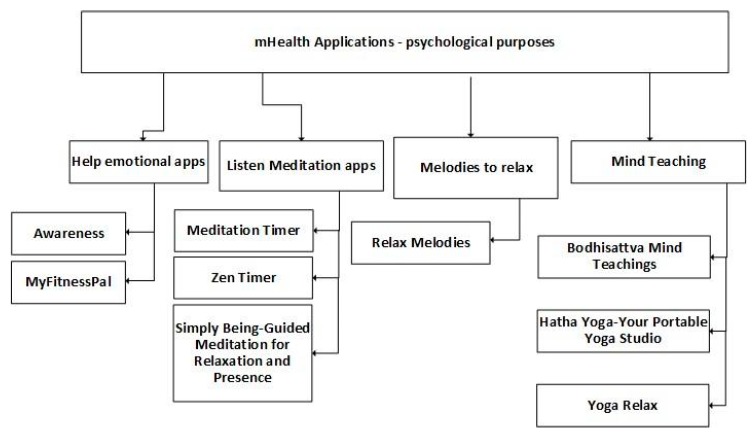
Classification of mobile health applications related to psychological purposes.

Psychology is one of the therapeutic areas for which technology is useful. Figure 6 presents the subcategories of the mobile applications previously analyzed, where most of them are related to music and meditation. These mobile applications provide several features, including help with emotions, listening to meditation sessions, relaxing melodies, and mind teaching. It is one of the types of mobile applications that can be accessed off-line, because the different songs, medication sessions, and teaching sessions may be available without Internet, and the various therapies do not need contact with healthcare professionals.

**Figure 7:**
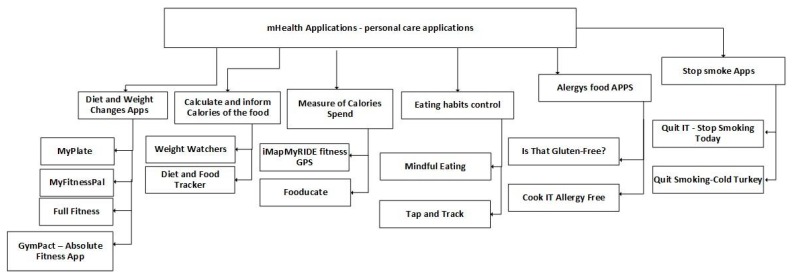
Classification of mobile health applications related to personal care.

Related to personal care, Figure 7 classifies the analyzed applications into six subcategories: diet and weight changes, calculate and inform calories of the food, measurement of calories spent, eating habits control, allergies food apps, and stop smoking applications. These mobile applications use the different sensors included in mobile devices and help people to maintain a healthy lifestyle. Commonly, an Internet connection is not necessary to use these applications, but it may be needed to check some values online. These applications can also act as a personal trainer.

**Figure 8:**
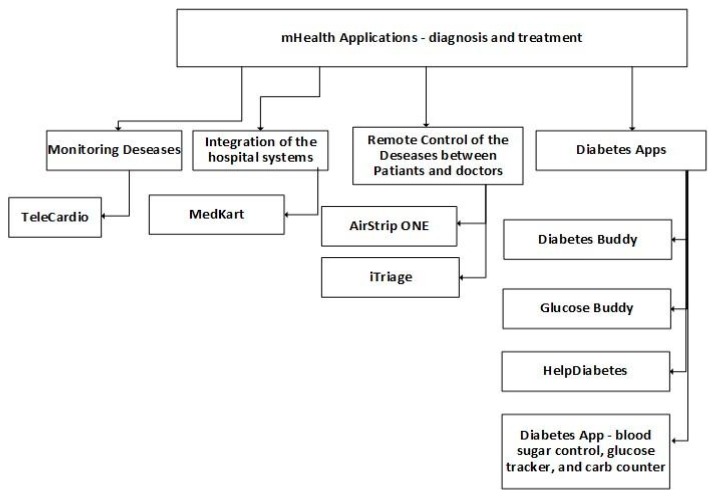
Classification of mobile health applications related to diagnosis and treatment.

In Figure 8, the subcategories of the mobile applications related to diagnosis and treatment include monitoring diseases, integration with hospital systems, remote control of the health disorders between patients and doctors, and diabetes applications. These applications require a constant Internet connection to provide communication features with healthcare professionals and the monitoring of different parameters related to specific diseases. The scientific validity of these applications is not available, which is significant since these mobile applications deal with critical parameters.

**Figure 9:**
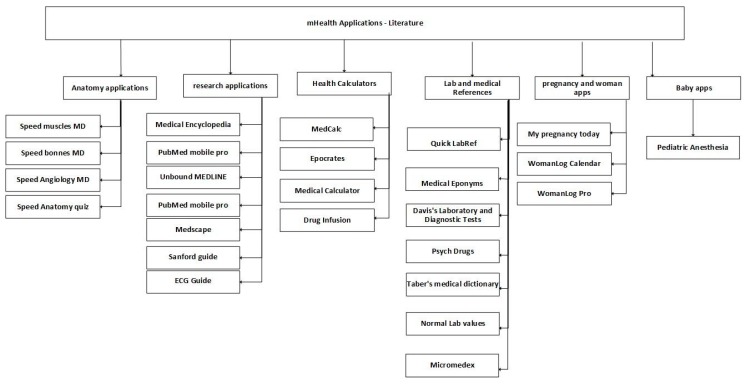
Classification of mobile health applications related to literature.

The mobile health applications related to literature incorporate different subjects of medicine. These applications support medical students and allow the consulting of innovative information. Moreover, these mobile applications promote the instruction of people in different situations. Consequently, Figure 9 presents six categories: anatomy applications, research applications, health calculators, lab and medical references, pregnancy and woman apps, and baby apps. These applications are useful in different situations, and, commonly, use information from validated sources.

## 6. Conclusions

Nowadays, the use of mobile devices has been increasing. These portable devices have several sensors available, such as accelerometer, gyroscope, proximity sensor, built-in GPS receiver, cameras, and microphone, including the possibility to connect other sensors via Bluetooth. The two platforms most used in the world are the Android operating system and the iOS operating system. The mobile applications can be useful in the healthcare area for patients and clinical staff. All mobile apps are available in online application stores.

Nevertheless, mobile health applications include several limitations, such as usability, ethics, network, and management. Firstly, usability includes the functions related to the mobile screen, data record, battery life, and contents. Secondly, ethics comprises privacy, trust, equity, and responsibility for errors [13,182,183,184]. Thirdly, network comprehends the bandwidth, integration, and coverage. Finally, management holds data protection, return on investment, authentication, and authorization. Furthermore, there is nowadays a trend to use blockchain in healthcare applications. It is a new technology, and, as a goal, it seems attractive. The participation of big data in public health is increasing, and it will be our future research goal. The most crucial future direction consists of the stress validation of mobile applications before they go to the market because it is a critical area.

This paper focuses on the analysis of mobile applications available on online application stores. External entities regulate these mobile applications. Most of the existing applications are literature applications and personal care applications, improving the quality of healthcare and patient’s diagnosis. Mobile health applications can enhance communication between healthcare professionals and patients by supporting telemedicine systems. The social networks for healthcare professionals allow the possibility of sending information to other professionals, asking questions about their patient’s problems, and sharing experiences with other people [185,186]. 

The use of mobile devices will continuously improve healthcare. Therefore, mobile health applications are a vital part of the relation between health and technology.

## Figures and Tables

**Table 1:** Mobile health applications related to literature applications.

Mobile Applications	Platforms	Free	Main Features
Speed muscles MD [67,68]	Android and iOS	No	Study of anatomy; test the speed and memory of identifying the muscles.
Speed bones MD [69,70]	Android and iOS	No	Study of anatomy; test the speed and memory of identifying the bones.
Speed Angiology MD [71,72]	Android and iOS	No	Study of anatomy; test the speed and memory of identifying the arteries and veins.
Speed Anatomy quiz [73,74]	Android and iOS	No	Study of anatomy; test the speed and memory of identifying the body’s anatomy.
Medical encyclopedia [75]	Android	Yes	Contains more than 50,000 pages of detailed medical information.
PubMed mobile pro [76]	Android	Yes	Provides a web interface adapted to mobile devices to access a PubMed library.
Unbound MEDLINE [77]	iOS	Yes	Provides a web interface simplified and adapted to mobile devices to access a PubMed library.
Medscape [78,79]	Android and iOS	Yes	Offers a huge drug reference library; offers a disease library, procedures, and protocols; offers a drug interaction checker with more resources, such as search and save articles searched for future reference.
MedCalc [80,81]	Android and iOS	No	Gives easy access to a wide array of medical formulas and scores.
Drug infusion [82]	iOS	No	Intravenous medication drip rate calculator; offers both weight-based and non-weight-based calculations with unit conversion flexibility.
Quick LabRef [83]	Android	Yes	Provides a quick look at the up-to-date information on the most commonly used clinical laboratory values and other useful relevant information.
Medical Eponyms [79,84]	Android and iOS	No	Allows for quick lookup, the meaning of more than 1700 medical eponyms using full-text search or by selecting from one of 28 categories.
Taber’s medical dictionary [85,86]	Android and iOS	No	Presents a medical dictionary used by healthcare professionals.
Sanford guide [87,88]	Android and iOS	Yes	Allows healthcare professionals who care for patients with infectious diseases to see their subscriptions.
Epocrates [89,90]	Android and iOS	Yes	Various purposes related to healthcare professionals.
My pregnancy today [91,92]	Android and iOS	Yes	Guide pregnant women about their pregnancy and answer their questions.
WomanLog Calendar [93,94]	Android and iOS	Yes	Shows a menstrual and fertility calendar for women, helping the women to know their fertile period.
Pediatric Anesthesia [95,96]	Android and iOS	No	Gives a maintenance rate; allows the user to input insensible and blood losses to generate an hourly total; gives the doses of oral ketamine, midazolam, and nasal dexmedetomidine; gives heart and respiratory rates and blood pressure appropriate by age; gives calculated doses.
ECG Guide [97,98]	Android and iOS	No	Teaches guide to ECG interpretation with examples.
Normal Lab Values [99,100]	Android and iOS	Yes	Shows reference values both in traditional and SI units; visualizes labs by categories or alphabetical list; enables to find values.
Medical Calculator [102]	Android	No	Includes various formulas and equations.
Davis’s Laboratory and Diagnostic Tests [103,104]	Android and iOS	No	Provides evidence-based information on the selection and interpretation of common laboratory tests.
Pocket Guide to Diagnostic Tests [105,106]	Android and iOS	No	Provides evidence-based information on the selection and interpretation of common laboratory tests.
Micromedex [107,108]	Android and iOS	Yes	Used for on-the-go access to the industry’s most trusted clinical reference information, providing users the peace-of-mind of knowing the information to the healthcare professionals.
Psych Drugs [109]	iOS	Yes	Shows information about various psychotropic medications, including antipsychotics, antidepressants, anti-anxiety medications, and mood stabilizers.

**Table 2:** Mobile health applications related to diagnosis and treatment.

Mobile Applications	Platforms	Free	Main Features
iTriage [115]	Android	Yes	Diagnosis of the health state of the patient; finds a healthcare professional to the problem in their location
Diabetes Buddy [117]	iOS	No	Helps people manage diabetes; tracks factors that influence the blood sugar level; monitor the fluctuations of blood sugar level; help users plan accordingly and helping people to share the data with their healthcare professionals.
Glucose Buddy [118,119]	Android and iOS	Yes	Tracks glucose readings entered four times a day, as well as food consumed, exercise, insulin dosage, and activities; allows sending this by e-mail.
HelpDiabetes [120]	iOS	Yes	Calculates the number of carbohydrates, tracking total fats and proteins.
Diabetes App - blood sugar control, glucose tracker, and carb counter [121]	iOS	No	Help patients to manage their diabetes; track the factors that influence their blood sugar level; monitor the fluctuations; plan accordingly; share their data with their healthcare professional.
Diabetes Lite App - blood sugar control, glucose tracker, and carb counter [122]	iOS	Yes	Help patients to manage their diabetes; track the factors that influence their blood sugar level; monitor the fluctuations; plan ahead accordingly, share their data with their healthcare professional.

**Table 3:** Mobile health applications related to personal care applications.

Mobile Applications	Platforms	Free	Main Features
MyPlate [123,124]	Android and iOS	Yes	Control the user’s diet, weight change, and workout to help people stay fit.
MyFitnessPal [125,126]	Android and iOS	Yes	Helps the user’s diet; control your weight and calories spent during your daily physical activities, store the results in a remote database.
Weight Watchers [128,129]	Android and iOS	Yes	Calculate the calories needed and use barcodes to insert the items and check your calories.
iMapMyRIDE fitness GPS [130,131]	iOS	Yes	Measures the calories spent, the time, and separates these values by types of activities.
Fooducate [132,133]	Android and iOS	Yes	Allow the weight loss and diet control.
Mindful Eating [134,135]	Android and iOS	No	Helps to build mindful eating habits over time, awarding badges for nutritional milestones and points out nutritional facts about food and helping users recognize patterns and provides tools for change.
Tap and Track [136]	iOS	No	Focuses on food, exercise, and weight; computes the nutritional intake on foods eaten, physical activity, current weight, and target weight; tracks the calories spent by calculating the BMR; finds the daily calorie count.
Is That Gluten-Free? [137,138]	Android and iOS	No	Application designed for those with gluten sensitivities, celiac disease, or anyone interested in gluten-free eating, including search tools by categories, brand and product name, filtering by safe, unsafe, and possibly unsafe ingredients.
Cook IT Allergy Free [139]	Android	No	Provides a library of recipes for those sensitive to gluten, dairy, eggs, and nuts, suggesting substitutions and offering the ability to customize recipes to avoid specific ingredients and add personal recipes in the “recipe box”.
Diet and Food Tracker [140]	Android	No	Track foods, calories, and weight; track exercises.
Full Fitness [141]	iOS	No	Provides a robust exercise database; provides clear illustrations, 175 videos, and instructions; allows the users to add exercises, track their progress, and e-mail the data.
GymPact–Absolute Fitness App [142]	Android	No	Allows users to log and manage their food intake, exercise, and weight, allowing the automatic calculation of daily nutrient limits based on personal profile and dietary goals.
Quit IT - Stop Smoking Today [143]	iOS	No	Motivate and encourage to quit; helps ex-smokers avoid relapse; track cigarettes not smoked, money saved, and the benefits of quitting.
Quit Smoking-Cold Turkey [144]	iOS	No	Help the user to stop smoking; calculates the number of cigarettes daily.

**Table 4:** Mobile health applications related to psychological purposes.

Mobile Applications	Platforms	Free	Main Features
Awareness [156]	iOS	No	Tool to intercepts peoples’ daily routines; prompts routines to get in touch with what they are feeling; brings routines into the present moment; provides insight and breaks patterns of emotions, attitudes, and behavior through awareness and inspirational practices.
Meditation Timer [146,157]	Android and iOS	No	Listen meditations that can be customized by name, time, and sound settings.
Relax Melodies [147,148]	Android and iOS	Yes	Offers 70 high-quality ambient sounds, volume adjustment for each sound, and binaural beats for brainwave synchronization to help induce relaxation, meditation, and sleep.
Zen Timer [149,150]	Android and iOS	No	Used to mindful activities.
Simply Being-Guided Meditation for Relation and Presence [151]	iOS	No	Used to choose voice-guided meditation length of 5, 10, 15, or 20 min with the option to listen with or without music or nature sounds.
Bodhisattva Mind Teachings to Cultivate Courage and Awareness in the Midst of Suffering by Pema Chodron [152]	iOS	No	Explores the insights and practices from the teachings of an eighth-century Buddhist classic, The Way of the Bodhisattva, on the key to true liberation, the taming of the mind.
Hatha Yoga-Your Portable Yoga Studio [153]	Android	Yes	Provides hours of professionally recorded instruction, including built-in classes for various skill levels, the ability to customize any class to suit individual needs, meditation and breathwork instruction, a pause feature, and the option to select the background music.
Yoga Relax [154]	Android	No	Shows yoga sessions, including the ability to turn on or off an audio instructor, detailed information about poses, the required steps for correct positioning, and how to maintain a pose.
Authentic Yoga with Deepak Chopra and Tara Stiles [155]	iOS	No	Contains video explanations of individual poses, breathwork, and other yoga practices with customizable routines, and a full explanation of Authentic Yoga.

**Table 5:** Mobile health applications related to specialty education.

Mobile Applications	Platforms	Free	Main Features
Electronic Preventive Services Selector (ePSS) [158]	iOS	Yes	Set of calculators and tools with simple screening tools for a variety of situations.
Radiology 2.0 [159]	iOS	Yes	Contains 65 radiology cases that pertain to emergency medicine.

**Table 6:** Mobile health applications to check PHR.

Mobile Applications	Platforms	Free
Capzule PHR [162]	iOS	No
Health and Family [163]	Android	No
HealthNotes [164,165]	Android and iOS	No
OnPatient Personal Health Record [167]	iOS	Yes
Patient [168,169]	Android and iOS	Yes

**Table 7:** Mobile health applications related to educational applications for patients.

Mobile Applications	Platforms	Free	Main Features
drawMD- Patient Education [170]	iOS	Yes	Allows healthcare professionals to draw out surgical procedures to their patients in an easy manner and contains mobile applications related to various areas of medicine.
First Aid [171,172]	Android and iOS	Yes	Provides information on urgent and emergent medical situations.
VueMe [173]	iOS	Yes	Used by patients for the non-diagnostic viewing of medical images and share it to a cloud service.
iMuscle 2 [174]	iOS	Yes	Used to identify a body part or individual muscle by zooming into a 3D human body with the musculature exposed and access to all the exercises.

**Table 8:** Mobile health applications related to social networking.

Mobile Applications	Platforms	Free	Main Features
Doximity [175,176]	Android and iOS	Yes	Help the healthcare professionals to find any other, wanting to communicate.
Univadis US [177,178]	Android and iOS	Yes	Aid healthcare professionals to learn and improve their practice and users can directly message other users outside of public forums, with interactions around topics in medical research, clinical care, policy, and regulation.
DocBookMD [179,180]	Android and iOS	Yes	Allows the communication between healthcare professionals.
